# *Azadirachta indica* A. Juss. In Vivo Toxicity—An Updated Review

**DOI:** 10.3390/molecules26020252

**Published:** 2021-01-06

**Authors:** Teresa M. Braga, Lídia Rocha, Tsz Yan Chung, Rita F. Oliveira, Cláudia Pinho, Ana I. Oliveira, Joaquim Morgado, Agostinho Cruz

**Affiliations:** 1 Centro de Investigação em Saúde e Ambiente, Escola Superior de Saúde, Instituto Politécnico do Porto, 4200-072 Porto, Portugal; lcar@ess.ipp.pt (L.R.); tycg@ess.ipp.pt (T.Y.C.); rfo@ess.ipp.pt (R.F.O.); clp@ess.ipp.pt (C.P.); aoliveira@ess.ipp.pt (A.I.O.); 2 Bio4Life4You, 4460-170 Porto, Portugal; jcmorgado@bio4life4you.com; 3 World Neem Organization, Mumbai 400101, India

**Keywords:** toxicity, in vivo, *Azadirachtaindica*

## Abstract

The Neem tree, *Azadirachta indica* A. Juss., is known for its large spectrum of compounds with biological and pharmacological interest. These include, among others, activities that are anticancer, antibacterial, antiviral, and anti-inflammatory. Some neem compounds are also used as insecticides, herbicides, and/or antifeedants. The safety of these compounds is not always taken into consideration and few in vivo toxicity studies have been performed. The current study is a literature review of the latest in vivo toxicity of *A.* *indica*. It is divided in two major sections—aquatic animals toxicity and mammalian toxicity—each related to neem’s application as a pesticide or a potential new therapeutic drug, respectively.

## 1. Introduction

Throughout time, plants have played an important role in treating different human diseases. Several plant species have, in one or more of its organs, substances that can be used for therapeutic purposes. According to the World Health Organization (WHO), these are called medicinal plants [1]. *Azadirachta indica*, commonly known as neem, an evergreen tree of the Meliaceae family, native from India, has been used for thousands of years in traditional systems of medicine, such as Ayurveda. From the various parts of neem, including leaves, bark, seeds, flowers, fruits, and roots, a large number of phytochemicals with different biological and pharmacological attributes, such as azadirachtin, nimbolide, gedunin, azadirone, salannin, and others, can be extracted [2,3,4,5,6]. These pharmacological activities include, but are not limited to, anticancer, antibacterial, antifungal, antiviral, antileishmanial, anthelmintic, antimalarial, antipyretic, analgesic, anti-inflammatory, antidiabetic, antiallergenic, neuroprotective, cardioprotective, and pesticidal activities [4,5,6]. Due to its therapeutic characteristics, the neem tree is also known, especially in India, as a “village pharmacy” [7] and was declared by the United Nations as “The tree of the 21st century” [8]. Although various parts of neem have an extensive use in traditional systems of medicine [4], the measurement of toxicities of its natural compounds is crucial before its application as a therapeutic drug [9]. Studies based on animal models confirmed that, at certain dosages, neem is safe, but on another hand, neem and its compounds may show toxic/adverse effects [9]. The safety of neem as a pesticide, and of its derivatives, also represents an important issue and was previously reviewed by Boeke et al. [10].

The present review aimed to provide a state-of-the-art analysis about *A. indica*’s in vivo toxicity, focusing on the safety evaluation in aquatic animals and mammalians. This manuscript represents, to our knowledge, an updated review on this topic.

## 2. Safety Evaluation

### 2.1. Aquatic Animals Toxicity

The contamination of aquatic environments represents a cause for concern. Its evaluation uses different methodologies based on several bioindicators, such as invertebrates, fish, and algae [11]. The use of invertebrates for toxicity tests presents some advantages as it reduces the number of mammals and space required, thus becoming less expensive. These organisms may also provide initial information on the pharmacologic applicability of nanoplatforms for drug delivery [12]. Fish models, namely the most commonly used zebrafish, are also easy to obtain, maintain, and reproduce in the laboratory [13]. Invertebrates present a limitation, as they have a different biological organization level relative to mammals and, so, their use should be considered as a prescreening method [14]. This limitation does not extend to fish models, as they are vertebrate and, thus, share basic nervous system organization with other vertebrates, such as humans [15].

#### 2.1.1. Acute Toxicity

Saravanan et al. [16] evaluated the acute toxicity of aqueous extracts of *A. indica* leaves in fish, *Cirrhinus mrigala*. Different doses of extract were used (0.25, 0.50, 0.75, 1.0, 1.25, 1.50 ppm) and, for each, ten fish were introduced and kept in separate glass tanks. Mortality was recorded after 24 h and the half maximal lethal concentration (LC_50_) value obtained was 1.035 ppm. Behavioral changes were also observed, such as hypersensitivity, erratic swimming, abundant secretion of mucus, air gulping, and loss of reflex [16]. Changes in hematological, ionoregulation, biochemical, and enzymological parameters of fish were also observed. Mwangi et al. [17] determined the safety of *A. indica* root bark aqueous extract using the Brine shrimp lethality test by exposing *Artemia salina* L. nauplii to different concentrations of the extract (10, 100, and 1000 µg/mL) for 24 h. The extract was considered moderately toxic, as LC_50_ obtained was 285.8 µg/mL. Zebrafish embryo toxicity to *A. indica* green callus methanolic extract was carried out by Ashokhan et al. [18], using six different concentrations ranging from 500–5000 µg/mL for 5 days. After treatment, LC_50_ value was determined to be 4606 µg/mL and the extract was considered nontoxic. Besides, zebrafish embryos treated with concentrations below 4000 µg/mL were able to hatch into larvae, while embryos treated with 5000 µg/mL presented a 0% hatching rate. Zebrafish larvae also presented a normal heart rate (136 beats/min) and no teratogenic defects were observed in embryos or larvae [18]. According to this study, green callus, obtained from *A. indica* leaf, has the potential to produce an environmentally and sustainable friendly pesticide.

Acute effects of commercial neem insecticides, Azatin (containing 3% azadirachtin (AZA)) and Neemix 4.5 (containing 4.5% of azadirachtin and 3–5% of neem oil), on *Daphnia pulex* were evaluated by Stark [19]. The aquatic invertebrates were exposed to different concentrations of each product for 48 h. The LC_50_ values obtained were 0.57 mg AZA/L (concentration based on azadirachtin equivalence) and 0.68 mg AZA/L for Azatin and Neemix, respectively, thus considered equitoxic. Acute toxicity of the other two neem-based insecticide, Neemix^TM^ (0.25% AZA) and Bioneem^TM^ (0.09% AZA), and pure AZA, were also determined for *D. pulex* [20]. The water fleas were exposed, for 48 h, to different concentrations of each compound (ranging between 0 to 0.5 µg AZA/mL) and the LC_50_ value was determined at the end of this period. Of the three compounds tested, Bioneem^TM^ presented the lowest LC_50_, 0.03 µg AZA/mL, followed by Neemix^TM^, 0.07 µg AZA/mL, and AZA with 0.382 µg/mL. Therefore, Bioneem^TM^ was the most toxic compound for *D. pulex* on this study [20]. Goktepe and Plhak [20] also evaluated the acute toxicity of AZA, Neemix^TM^, and Bioneem^TM^ on *Procambarus clarkii*. This species was exposed to different concentration of each compound for 96 h. This crustacean was less sensitive to both pesticides (LC_50_ of 4.71 and 6.60 µg AZA/mL for Neemix^TM^ and Bioneem^TM^, respectively) than to pure AZA (LC_50_ > 1 µg/mL). The acute toxicity of these pesticides was also studied, after 96 h of exposure, in other crustaceans, such as *Penaeus setiferus*, *Palaemonetes pugio,* and *Callinectes sapidus* [20]. From these, *P. pugio* was the least sensitive to both compounds (LC_50_ of 3.19 and 3.81 µg AZA/mL for Bioneem^TM^ and Neemix^TM^, respectively), followed by *P. setiferus* and *C. sapidus*, with LC_50_ of 2.68 and 1.15 µg AZA/mL for Neemix^TM^, respectively. These authors [20] also used the third instar larvae of mosquitoes (*Culex quinquefasciatus*) to test the acute toxicity of Neemix^TM^ and Bioneem^TM^ in a 96 h exposure test. Both pesticides had strong toxicity against these larvae, with LC_50_ values of 0.57 and 0.14 µg AZA/mL for Bioneem^TM^ and Neemix^TM^, respectively. The acute toxicity of Bioneem^TM^ was also tested on *Ceriodaphnia dubia* by Botelho et al. [21]. The animals were exposed to different concentrations of the product (0.01, 0.1, 1, and 10 mL/L) for 48 h. After that, the LC_50_ calculated was 0.032 mL/L, revealing toxicity to the organisms tested.

Pereyra et al. [22] tested the toxicity of Neem’s solution (a mix of 60% pure neem’s oil and 40% fatty ethoxylated alcohol of 9 mols as emulsifier) against *Limnoperna fortune* (larvae and adults), *Daphnia magna,* and *Cnesterodon decemmaculatus*. All tests were made with at least five concentrations (not specified) and one control. In the case of *D. magna*, the animals were exposed for 48 h and the LC_50_ value obtained was 17 µL/L. *C. decemmaculatus* were exposed to Neem’s solution for 96 h and the LC_50_ value reported was 4.92 µL/L. For the toxicity of *L. fortune*, larvae and benthonic adults (7, 13, and 19 ± 1 mm) were used and mortality was checked after 72 h. Larvae were more vulnerable to the toxicity than adults, and presented a LC_50_ value of 8 µL/L. The LC_50_ of adults changed with their size, 241, 249, and 122 µL/L for 7, 13, and 19 mm length, respectively. From the different species used in this study, adults of *L. fortune* were the least sensitive to Neem’s solution [22]. Thus, when used as a pesticide in agriculture, Pereyra et al. [22] recommended that it should not be used in open waters, but only in closed systems, in man-made facilities.

The acute toxicity of Bioneem oil (90% neem oil and 10% emulsifiers and synergetic ingredients) was tested against *D. magna* and *D. rerio*, for 48 and 96 h of exposure, respectively [23]. In the case of *D. magna*, the organisms were exposed to different concentrations of Bioneem oil, 0.015, 0.031, 0.065, 0.125, 0.250, 0.50, 1.0, 2.0 mL/L, and the median effective concentration (EC_50_) for immobility was determined, 0.17 mL/L. For *D. rerio*, the concentrations used were 0.16, 0.2, 0.32, 0.4, and 0.8 mL/L and LC_50_ value obtained was 0.22 mL/L. Maranho et al. [23] concluded that *D. magna* had higher sensitivity to the bio-insecticide studied than *D. rerio*.

#### 2.1.2. Chronic Toxicity

Botelho el al. [21] studied the chronic toxicity of Bioneem^TM^ in *C. dubia*. These animals were exposed to different concentrations of the product (0.001, 0.002, 0.004, 0.008, and 0.016 mL/L) for 7 days. After this period, a decrease in the number of neonates per female at higher concentrations was observed. Thus, Bioneem^TM^ presented a toxicity effect in the reproduction of *C. dubia*. Maranho et al. [23] evaluated the chronic toxicity of Bioneem oil in *D. magna*. In a 21-day assay, five different concentrations were used, 0.0106, 0.0212, 0.0425, 0.0850, 0.17 mL/L, and, at the end of the test, the average number of neonates produced and the sizes of adult organisms were compared between the control and treatments. This chronic test revealed some Bioneem oil effects, such as reduced number of neonates and inhibition of size. It was also possible to observe that means of both parameters were significantly lower when compared to the control, consequently revealing a toxic effect [23]. Taking these results into consideration, and also the ones obtained from the acute toxicity tests, Maranho et al. [23] concluded that even at low concentrations Bioneem oil may cause adverse effects to aquatic organisms.

#### 2.1.3. Genotoxicity

The genotoxic effects of azadirachtin were assessed in fish, *Oreochromis mossambicus,* by Chandra and Khuda-Bukhsh [24]. Animals were injected intramuscularly, with 0.005% of azadirachtin at 1 mL/100 g body weight (b.w.), kept separately, and fed with a standard diet until sacrificed (6, 24, 48, 72, and 96 h after treatment). Somatic metaphase complements were carefully observed for any structural and numerical alteration and, as result of the treatment, changes were detected, such as break, terminal association, centric fusion, precocious centromeric separation, and C-mitosis [24]. In terms of proteotoxicity, azadirachtin treatment drastically reduced the number of protein bands (on electrophoretic gel), particularly in tissues such as kidney, dorsal muscle, and gill. Quantitatively, azadirachtin also induced changes in the protein content of tissues, such as kidney, liver, and spleen (mild changes) and gill and heart (drastic changes). Thus, Chandra and Khuda-Bukhsh [24] suggested that the use of this natural product, azadirachtin, should be viewed with caution.

It is important to note that in real conditions, other chemical and microbial activities would also influence the effect of such compounds/pesticides [20]. Table 1 summarizes the different studies of toxicity of neem using different aquatic animals. In most studies acute toxicity assays were performed and neem pesticides or derivatives were tested. Concluding from these studies, neem extracts presented the lowest toxicity in acute tests. Chronic and genotoxicity assays revealed toxicity to the animals tested.

### 2.2. Mammalian Toxicity

These animal models have the advantage of sharing certain characteristics with humans, which are lacking in some other models, such as placentation, development in utero, and maternal/fetal metabolism and interactions [25]. The mammalian models organisms mainly include mice, rats, and rabbits.

#### 2.2.1. Acute Toxicity

Aqueous extracts of *A. indica* leaves were evaluated for acute toxicity by Dorababu et al. [26]. This study was carried out in mice, using different doses of extracts (200, 500, 1000, and 2500 mg/kg b.w.) and observing the animals for 24 h. No mortality was observed and the median lethal dose (LD_50_) value was considered higher than 2500 mg/kg. A similar study was performed by Kingsley et al. [27], who orally administrated different doses of aqueous extracts of *A. indica* to mice (1250, 2500, and 5000 mg/kg b.w.). No obvious toxic side effects were observed and treated mice were found healthy and normal with no record of weight or hair loss, allergy, or other symptoms of discomfort. Thus, the LD_50_ value was considered higher than 5000 mg/kg. The acute toxicity of aqueous extracts of *A. indica* leaves and seeds were tested in rats at different concentrations, 0.05, 0.071, 0.084, 0.092, and 0.1 g/mL and 0.1, 0.15, 0.17, and 0.2 g/mL, respectively [28]. The study was performed for 2 days by intramuscular injection of the animals with the different concentrations of the extracts (0.07 mL/g b.w.). Aqueous extracts of leaves and seeds presented a LD_50_ value of 6.2 and 9.4 mL/kg, respectively, thus presenting acute toxicity when injected in rats.

A suspension of ethanolic extract of *A. indica* leaves was orally administered at 5 g/kg b.w. dose to mice, for 7 days [29]. No acute toxicity manifestation or death were observed, thus the LD_50_ value was considered higher than 5 g/kg. Kanagasanthosh et al. [30] also evaluated the acute toxicity of ethanolic extract of *A. indica* leaves, but for 14 days in rats. During treatment, oral doses of 20, 200, or 2000 mg/kg b.w. of extract were administered to the animals. After treatment, no significant changes were observed in the behavioral or autonomic responses of the animals, and no mortality was registered. Thus, the LC_50_ was considered higher than 2000 mg/kg b.w. Achi et al. [31] orally administered different doses of ethanolic extracts of *A. indica* leaves to mice (50 to 5000 mg/kg b.w.) and observed them for 1 day. No mortality occurred, so LD_50_ was considered higher than 5000 mg/kg and the extract was considered safe. A similar assay was performed by Oseni and Akwetey [32] and LD_50_ value was considered higher than 1000 mg/kg. Recently, similar results were obtained by Tepongning et al. [33], who evaluated the acute toxicity of hydroethanolic extracts of *A. indica* leaves using rats. During 14 days, oral doses of 2000 and 5000 mg/kg b.w. of extract were administered and LD_50_ was considered higher than 5000 mg/kg b.w., as no significant behavioral changes or morbidity/mortality were observed [33]. Thus, it was classified as practically nontoxic and without any risk to human health. During these 14 days of testing, the animal body weight did not significantly increase, with a maximum weight gain of 2.6 g for the treated groups compared to 2.7 g in the control group. Akin-Osanaiye et al. [34] administered, to mice, methanolic extract of *A. indica* leaves at doses of 10, 100, and 1000 mg/kg b.w., via the intraperitoneal route, and observed the animals for signs of toxicity and death for 24 h. Animals exhibited slow movement within the first six hours of administration and, after 24 h, concentrations of 100 and 1000 mg/kg of extract produced mortality. The LD_50_ obtained was 31.62 mg/kg and the extract was classified as highly toxic [34].

Acute toxicity tests of crude ethanolic extracts of *A. indica* stem bark were carried out in rats by Mbaya et al. [35]. The animals were treated intraperitoneally with graded doses (100, 200, 400, 800, 1600, 3200, and 64,000 mg/kg b.w.) and observed for 24 h. Animals administered with the three highest doses manifested clinical signs such as depression, dehydration, malaise, anorexia, respiratory depression, coma, and death, with the symptoms being dose related. The LC_50_ value calculated was 870 mg/kg. Stem bark extracts were also tested by Akin-Osanaiye et al. [34], but in mice. In their study, methanolic extracts of this part of *A. indica* were administered at doses of 10, 100, and 1000 mg/kg b.w., via the intraperitoneal route, and the animals were observed for signs of toxicity and death for 24 h. The LD_50_ value obtained was 489.90 mg/kg and the extract was classified as moderately toxic [34].

The acute toxicity of another part of *A. indica*, flowers, has also been tested in rats. Kupradinum et al. [36] orally administered different concentrations of methanolic extracts of *A. indica* flowers (6, 9, and 12 g/kg b.w.) for 14 days, and no signs or symptoms of toxicity were observed. Thus, the LD_50_ was considered higher than 12 g/kg.

Neem oil acute toxicity was determined by Deng et al. [37] in a 14-day assay. In this study, mice were orally treated with neem oil mixed with 1% carboxymethyl cellulose at the doses of 18.40, 23.00, 28.80, 36.00, and 45.00 g/kg. In each case, the product volume administered by gavage was 2 mL/100 g b.w. Fifty minutes after treatment, mice treated with higher doses appeared to move slowly, were very sensitive to noise, and got chills and convulsions. Death necropsy revealed a lot of liquid filling and intestinal swelling in the guts of mice, while other tissues and the organs showed no obvious abnormalities. The LD_50_ value obtained was 31.95 g/kg and Deng et al. [37] classified the neem oil as not toxic.

A study based on rabbits was performed to check the toxicological analysis of aqueous extract of *A. indica* leaves [38]. Extracts were administered orally daily, by gavage, to rabbits for 14 days at dose levels of 5 or 24 mg/kg b.w. No signs of toxicity were observed, although animals became dull and lost appetite. A progressive increase in body weight, in both test and control animals, was observed. No acute toxic effect was observed on hematological and biochemical parameters, and gross and histopathological lesions detected did not reveal any significant pathology to any of the organs. Boadu et al. [38] considered 2.5 mg/kg the no-observed-adverse-effect-level (NOAEL) of crude aqueous extract of neem leaves in rabbits.

Table 2 summarizes the different studies of acute toxicity of neem in mammals. It can be said that the acute toxicity of neem is more related to the mode of administration of the extract/sub product, than to the part of the plant, extract, and/or concentration. Animals only revealed acute toxicity when treated by intramuscular injection or via the intraperitoneal route. When treated orally, no significant changes or toxicity were recorded.

#### 2.2.2. Subacute Toxicity

Dorababu et al. [26] studied the subacute toxicity of aqueous extracts of *A. indica* leaves, at 1000 mg/kg b.w., by giving it orally to rats for 28 days. After treatment, no mortality was registered, nor histological changes in the kidney, liver, testis, or adrenals, but their weights increased or tended to increase. In additional, hematological parameters and liver and kidney function tests revealed few or no change from the control values, indicating *A. indica* leaf extract as a safe drug. Haque et al. [39] tested the toxicity of aqueous extracts of *A. indica*, in what they called neem leaf preparation (NLP). According to these authors, extracts obtained from 0.25, 0.5, and 1 mg of dry neem powder were considered as 0.5, 1, and 2 units of NLP, respectively. Mice were treated with 0.5, 1, or 2 units of NLP weekly for 28 days. After treatment, no mortality occurred at the lowest tested concentrations. However, at 2 units of NLP, the mice’s survival was 83% and 66% after second and third week of treatment, respectively. Surviving mice, of all tested concentrations, showed no significant changes in physical behavior, body weight, or organ to body weight ratio. NLP treatment stimulated hematological systems, as evidenced by the increase in total count of red and white blood cells, platelets, and hemoglobin percentage. Due to their results, Haque et al. [39] considered NLP as of nonhepatotoxic nature. Mallick et al. [40] evaluated the toxicological profile of neem leaf glycoprotein (NLGP) in both mice and rats. Different doses of NLGP (25, 50, 100, and 200 μg) were weekly injected in the animals for four weeks. No death nor behavioral changes were observed in any animal, as well as no significant changes in body or organs weight. Besides, injected mice showed more stretching and/or jumping gestures that the control mice, without being considered hyperactive. Both histological evaluation of brain, kidney, lung, liver, spleen, and lymph nodes and the hematological profile of injected animals were normal. Kidney and liver functions were not affected. Additionally, a small hematostimulation was noticed, as shown by increased hemoglobin content, lymphocyte numbers, and leukocyte count. In both mice and rats, no apoptotic effect on immune cells was observed, but NLGP induced proliferation of mononuclear cells collected from the animals. This study, also confirmed that NLGP had no killing effect on immune cells and it maintained the immune functions in activated state, but not hyperactivation [40].

The subacute toxicity of ethanolic extracts of *A. indica* leaves were tested for 28 days, with oral doses of 75, 150, and 300 mg/kg b.w. daily administrated [33]. No changes were observed in the animals during treatment and the rats grew normally, in both treatment and control groups, with a total weight gain of approximately 10 to 20 g. The biochemical parameters profile, after treatment, revealed no significant changes (*p* > 0.05), with the exception of the aspartate amino transferase (ASAT) level of animals treated with 300 mg/kg b.w., which increased when compared to the control, 213.58 and 153.85 U/L, respectively [33]. This suggests muscular dysfunction or damage to internal organs, which was confirmed by autopsy of the animals on day 28. A general darkening of the liver with the presence of nodules in animals treated with extracts at 300 mg/kg b.w., and the presence of lung nodules in one animal treated with extract at 150 mg/kg b.w., compared to the controls, was observed [33]. Although, no differences in weights of organs such as hearts, lungs, kidneys, livers, and spleens, were registered.

Ethanolic extract of *A. indica* stem bark at 50, 100, 200, and 300 mg/kg b.w. was orally administered to rats for 21 days [41]. After treatment, and specially at higher doses, the levels of white blood cells, platelets, serum triacylglycerol, and high-density lipoprotein cholesterol decreased significantly (*p* < 0.05). Although, the final body weights; absolute weights of the heart, lungs, liver, and kidney; organ–body weight ratios; total and conjugated bilirubin; low-density lipoprotein cholesterol; serum globulins; serum cholesterol; and computed atherogenic index increased significantly (*p* < 0.05). Changes in spleen–body weight ratio, alanine and aspartate transaminases, alkaline phosphatase, calcium, sodium, potassium, and food and water intake were also observed at specific doses. According to Ashafa et al. [41], only the dose of 50 mg/kg b.w. appeared to be relatively safe.

Bansod et al. [42] found no significant changes in body and pancreas weight and plasma biomarker levels in mice treated with nimbolide when compared to the control. In this study, mice received 1 mg/kg b.w. of nimbolide, per day, for 21 days. The compound was dissolved in dimethyl sulfoxide and intraperitoneally injected. The treatment also did not cause alterations in oxidative stress parameters, such as malondialdehyde, glutathione, and nitrite levels, as well as any changes in cytokines levels. After treatment, the architecture of pancreatic tissue was considered normal.

The subacute oral toxicity of neem oil was evaluated over 28 days by Deng et al. [37]. During this time, mice received a daily dose of 177, 533, or 1600 mg/kg b.w. After treatment, no death or effects on body weight were observed, as well as no significant differences, compared to the control, on serum biochemistry parameters of mice (*p* > 0.05). Also, the organ coefficient of liver, heart, lung, kidney, and spleen in treated mice presented no statistical difference compared to those of the control group (*p* > 0.05). Although, mice treated with a higher dose (1600 mg/kg) showed treatment-related clinical signs, such as loss of appetite and rough fur in the last 2 weeks, and histopathological examination revealed this dose had varying degrees of damage on kidneys, liver (consistent changes in both sexes of mice), and testicles.

Table 3 summarizes the different studies of subacute toxicity of neem. It can be inferred that the subacute toxicity of neem is more related to the concentration tested, as higher concentrations seem to be more toxic, than to the mode of administration of the extract/subproduct or part of the plant and/or extract. In general, subacute toxicity or significant effects were observed in animals treated with higher concentrations of the neem extract/subproduct.

#### 2.2.3. Subchronic Toxicity

Rahman and Siddiqui [43] studied the biochemical effects of vepacide on rats during subchronic exposure. Vepacide is a neem-based-pesticide, obtained by the isolations of a free-flowing solid from seed kernels of *A. indica*. Different doses of Vepacide in coconut oil (80, 160, and 320 mg/kg b.w.) were orally administered for 45 or 90 days. During treatment (45 days) and at the end of the treatment (90 days), an increase in acid (AcP) and alkaline (AkP) phosphatase in serum, kidney, lung, and liver tissue (AkP only in liver) and a decrease of AcP in liver were observed in cases where moderate and high doses were used. These changes were dose- and time-dependent and indicated that lung tissue was the most susceptible, followed by liver and kidney [43]. Although, 28 days post-treatment (withdrawal study) animal recovery was observed, indicating reversal of the toxic symptoms once the toxicant was removed.

Kupradinum et al. [36] fed rats with methanolic extract of *A. indica* flowers in 0.5% tragacanth daily for 90 consecutive days at the doses of 150, 750, and 1500 mg/kg b.w. After treatment, growth rate of male rats was affected, but no differences were recorded in both male and female rats’ weights. Histopathological examination of visceral organs also showed no change and blood chemistry values of most animals were within normal ranges. Although, in high-dose male rats, the levels of aspartate aminotransferase and blood urea nitrogen were significantly lower and creatinine was higher than in control rats. Alkaline phosphatase, creatinine, and potassium values were significantly higher in the female group receiving 750 mg/kg b.w. dose. Kupradinum et al. [36] concluded that the extract tested showed slight toxicity to rats at doses greater than 150 mg/kg/day.

Wang et al. [44] studied the subchronic toxicity of neem oil in mice, in order to determine the NOAEL of exposure and target organs of neem oil. For 90 days, neem oil was daily orally administered to mice in different doses, 177, 533, or 1600 mg/kg b.w. After treatment, no death or effects on body weight were observed, as well as no significant differences, compared to the control, on serum biochemistry parameters of mice (*p* > 0.05). Also, the organ coefficient of liver, heart, lung, kidney, and spleen in treated mice presented no statistical difference compared to those of the control group (*p* > 0.05). Although, mice treated with higher doses (1600 mg/kg) showed treatment-related clinical signs, such as loss of appetite and rough fur in the last 2 weeks, with very significant decreased in months two and three (*p* < 0.01). The histopathological examination revealed that this dose had varying degrees of damage on each organ except heart, uterus, and ovary (consistent treatment-related histopathological changes in both sexes). The damages observed were restored after the discontinuation of treatment for 30 days. Wang et al. [44] considered 177 mg/kg the NOAEL and that the target organs of neem oil toxicity were the kidneys, liver, and testicles.

Table 4 summarizes the three studies of subchronic toxicity of neem. It reveals that the subchronic toxicity of neem was dose dependent, higher concentrations were revealed to be more toxic, and its effects could be reversed after treatment discontinuation [43,44].

#### 2.2.4. Reproduction and Teratogenicity

Ghodesawar et al. [45] orally administered, to male rats, 100 mg of *A. indica* leaf powder in 1 mL of distilled water, every day, for 24 days, in order to elucidate its effect on the ultrastructural organization of the epithelial cells of cauda epididymis. Several ultrastructural changes were observed, indicating that the principal cell and the clear cell were affected, thus altering the composition of epididymal fluid, which may cause sperm mutations. A similar study was carried out by Aladakatti and Ahamed [46], who orally administered, to male rats, 100 mg of *A. indica* leaf powder in distilled water, every day, for 48 days, in order to detect changes in Sertoli cells. After treatment, bridges between Sertoli cell–germ cells and Sertoli cells–Sertoli cells were disturbed, coupled with changes in the Sertoli cells and cytoplasm, along with its organelle, such as damaged tubules and abundance of vacuoles.

Aladakatti et al. [47] orally administered different concentrations of aqueous extracts of *A. indica* leaves (125, 250, and 375 mg/kg b.w.) to male rats for 24 days. Dose-related effects on biochemical parameters of testis and epididymis were reported, suggesting that *A. indica* leaf extract has one or more constituents that may affect the androgen synthesis and, thus, exhibit antiandrogenic effects on androgen-sensitive target glands, such as testis and epididymis [47]. In order to determine whether aqueous extract of *A. indica* leaves induces generation of reactive oxygen species (ROS) and apoptosis through mitochondria-mediated pathway in rat oocytes, sexually immature female rats were fed palatable doses of extract (10 mg/g dry feed palate) for 10 days [48]. After that, rats were subjected to superovulation induction protocol. After treatment, morphological apoptotic changes were observed, associated with increases in hydrogen peroxide, nitric oxide, and cytochrome c concentrations; caspase-9 and caspase-3 activities; and DNA fragmentation in oocyte. Thus, the extract induces ROS generation leading to oocytes apoptosis via a mitochondria-mediated pathway.

Ethanolic extract of *A. indica* leaves at different concentrations, 65, 135, and 200 mg/kg b.w., were orally administered to female rats during the 4th, 5th, and 6th day of pregnancy and after birth (lactating females) for 15 days [49]. After treatment, there were no signs of systemic or reproductive toxicity, such as weight loss, diarrhea, ataxia, piloerection, stereotypes, vaginal bleeding, coma, or death. No neonatal malformation was observed upon external examination of the offspring from the control or experimental groups. The development of the nervous system and physical development of the offspring was not affected, as well as the body mass weight. Thus, the extract used did not cause any systemic toxicity, nor induced teratogenicity, being considered safe for use during the pre- and postnatal period in rats [49].

Terpenoid extract from the leaves of *A. indica* was orally administered to pregnant rabbits at 300 mg/kg b.w. (per day), for 11 days, starting from day 14 of the gestation period of the animals [50]. Lead acetate (50 mg/kg b.w.) was also administered to each animal after the extract. The results reported showed that terpenoid extract was well tolerated by pregnant animals, as no death, abortion, or stillbirth were observed. Also, the litters did not show any deformity or malformation. The animals pretreated with the extract had no statistically significant difference in lead concentration when compared to the lead-only treated group. Thus, Babalola and Areola [50] concluded that the terpenoid extract was nonembryotoxic, nonteratogenic, and nonabortifacient, but had no ability to interfere with lead from crossing the placenta and to reduce lead burden in the pregnant rabbits.

Raji et al. [51] studied the effect of methanolic extract of *A. indica* stem bark in male rats. Some animals were treated daily, by gavage, with 150 mg/kg b.w. of the extract and others with the same amount of extract plus vitamin E (100 mg/kg b.w.), both for 45 days. After treatment, there was no significant change in body and organ weights, nor in sperm volume of the animals treated with the extract, compared to the control. However, there was a significant decrease (*p* < 0.05) in sperm viability, serum testosterone level, and on lipid peroxidation and superoxide dismutase activity. In this study, it was also reported that vitamin E had a positive impact in the adverse effect caused by *A. indica* stem bark extract.

Ethanolic extract of *A. indica* seed was orally administered to rats at a 200 mg dose, in order to study its effect on estrous cycle, ovulation, and fertility [52]. For that, one group of animals was treated with extract dose for 3 weeks, another at 9 a.m. and at 6 p.m. on proestrus and on the morning of estrus, and another on day 1 to 5 postcoitum. After treatment, rats showed alterations in the estrous cycle, particularly a prolonged diestrus pattern and a partial block in ovulation. However, no anti-implantation/abortifacient effect was observed, as well as no teratogenicity in the fetuses. Seed extract of *A. indica* was also tested for its effect on follicular development in cyclic female albino rats by Roop et al. [53]. Polar and nonpolar fractions of the extract were orally administered at 3 and 6 mg/kg b.w. to the animals for 18 days. Results showed, for both fractions, a significant reduction (*p* < 0.05) in the number of normal single-layered follicles, follicles in various stages (I-VII) of follicular development, and total number of normal follicles. A similar study was carried out by Gbotolorun et al. [54], but using ethanolic extract of *A. indica* flowers. In order to analyze the effect on estrous cycle, rats received 1 g/kg b.w. of extract by gavage for 21 days. For the study of the effect on ovulation, one group of rats received 1 mg/kg b.w. of flower extract orally at 9 a.m. on proestrous and another group at 6 p.m. on proestrous. To understand the effect on fetus, one group of rats received 1 mg/kg b.w. of extract by gavage from day 1 to 5 postcoitum. After treatments, all animals fed with the extract had diarrhea and a 6.46% decrease in body weight. Eighty percent of the rats form the estrous cycle group test presented an irregular pattern of this cycle and also a prolonged diestrus pattern in each cycle. The effect on ovulation was only detected in the group of rats who received the extract at 9 a.m., with reduction in the number of ova shed in the oviduct. In this study, either pregnancy or the offspring were affected by the *A. indica* flower extract.

Different doses of aqueous wood ash extract of *A. indica*, 5, 50, and 100 mg/kg b.w. were orally administered to male rats in order to evaluate its reproductive toxicity [55]. Results showed no toxic effect on testicular weight and hormones (testosterone, follicle-stimulating hormone, and leuitinizing hormone). However, a significant decrease (*p* < 0.05) in sperm count, sperm motility, and live/dead sperm, as well as a significant increase (*p* < 0.05) of abnormal sperm were registered, which indicates that infertility occurred. The extract also presented toxic effect on spermatogenesis, proven by the histopathological changes in the testis observed, such as numerous apoptotic cells, vacuolation, and necrosis of the late elongated spermatids and seminiferous tubules with formation of multinucleated giant cells.

Srivastava and Raizada [56] reported absence of any major adverse reproductive effects in adults rats, as well as in 21-day-old pups of F_2B_ generation. In this study, the female rats were fed 100, 500, and 1000 ppm technical azadirachtin through diet, which is equivalent to 5, 25, and 50 mg/kg b.w. No toxicological effect was reported for parent rats or teratogenic effects in the F_1_ and F_2_ generations. There was also no evidence of cumulative effects on postnatal development and reproductive performance over two generations.

Dallaqua et al. [57,58] performed different studies in order to assess the effects of *A. indica* on lipid profile, oxidative stress status, and glycemic status in both nondiabetic and mildly diabetic pregnant rats [57] and on the frequency of congenital malformations in fetuses from pregnant rats [58]. In both studies, animals were treated with either neem seed oil (1.2 mL/day) or azadirachtin (1.0 mg/mL/day), orally administered throughout pregnancy for 21 days. In the first study [57], the treatment with azadirachtin and neem seed oil: (i) did not affect the lipid profile in nondiabetic dams; (ii) increased the proportion of fetuses classified as small for pregnancy age; (iii) had no hypoglycemic and anti-hyperglycemic effects on nondiabetic and diabetic rats, respectively; (iv) interfered with oral glucose test tolerance glycemic levels in diabetic rats; and (v) increased lipoperoxidation, characterized by increased malonaldehyde levels in nondiabetic rats. In the second study [58], no significant changes in glucose levels or total area under the curve were observed in animals treated with azadirachtin or neem seed oil. Although, an increment on the frequency of malformation/variation, in particular the visceral in fetuses, was shown in animals treated with neem seed oil. Thus, when administered during pregnancy, neem seed oil caused abnormalities in rat fetuses, showing a teratogenic effect [58], and both neem seed oil and azadirachtin impaired intrauterine development and altered antioxidant/oxidative status [57]. Another study evaluated the toxic and teratogenic, short- and long-term, effects on fetuses and pups of different doses (0.90, 1.80, 3.0, 9.0, and 45.0 mg/kg) of azadirachtin of neemix-4.5 (neem-based insecticide), orally administered on days 7–12 of gestation or during an 80-day period in pregnant mice [59]. No morphological or skeletal changes were produced in fetuses and pups.

Aladakatti et al. [60] daily administered subcutaneously, to male albino rats, different doses of azadirachtin-A (0.5, 1.0, and 1.5 mg/kg b.w.) for 24 days. After treatment, no significant differences were observed in animals’ body weight. However, the higher dose provoked a general decrease in reproductive organs weights, changes in biochemical parameters, and reduction in the sperm functional parameters, increasing abnormal sperms. Similar results were obtained in a 24-day assay, where nimbolide was administered subcutaneously to male albino rats at different concentrations, 0.5, 1.0, and 1.5 mg/kg b.w. [61]. After treatment, no statistically differences between animals’ body weights were observed, but their weights of reproductive organs decreased. The treatment also reduced sperm functional parameters and increased abnormal sperm counting in a dose-dependent manner. Biochemical analyses revealed a decrease in acid phosphatase and total protein content, and an increase in the total free sugar and activities of lactate dehydrogenase and alkaline phosphatase [61].

Table 5 summarizes the different studies of reproduction and teratogenicity of neem. In the majority of the studies reported, changes were described for both the female and male reproductive system, namely female and male gametes.

When performing in vivo studies it is important to have in mind that protocols should minimize the number of animals used, as well as their suffering [62]. Thus, replacing in vivo studies by in vitro studies should be considered, but these are only relevant if the cells used represent a target organ for the compound in study, and realistic concentrations are used. In fact, regarding *A. indica*, few in vitro studies have been performed. These included the evaluation of nimbolide, gedunin, and neem oil cytotoxicity on different normal cell lines. Nimbolide presented no toxicity against RWPE-1 cells, a normal prostatic cell line [63]; NIH3T3 cells, a mouse embryonic normal fibroblast cell line; and CCD-18Co cells, a colon normal fibroblast cell line [64,65]. This compound also presented no significant changes when tested for subacute effects in vivo [42]. Although nimbolide had some reproductive effects in rats, as abovementioned. In the case of neem oil, two in vitro studies found no significant effects in NIH 3T3 cells and CCD-18Co [66] cells, nor in MCF-10A cells—normal human mammary epithelial cells [67]—when low concentrations were used. The same was observed in the acute, subacute, and subchronic in vivo toxicity studies abovementioned using neem oil. This only had siginifican effects in high doses or in the reproduction/teratogenicity studies. Regarding gedunin toxicity, there are no recent in vivo studies, but low toxicity was described in vitro for adult-derived normal cells, Hs578Bst cells and human mammary epithelial cells (HMEs) [68], normal pancreatic cells (hTERT-HPNE) [69], human peripheral blood mononuclear cells [70], and normal human lung fibroblast (MRC-5) cells [71].

## 3. Material and Methods

A literature search using several online databases, such as PubMed, Science Direct, ISI Web of Knowledge, and Google scholar, was conducted up to October 2020. The principal search topics were related to *A. indica* and toxicity: acute toxicity, subacute toxicity, subchronic toxicity, and chemistry. Secondary searches included articles cited in sources identified by the previous search.

## 4. Conclusions and Future Perspectives

From the studies presented and highlighted in this review, it is clear that for aquatic organisms, neem extracts presented the lowest toxicity, while neem pesticides or derivatives presented moderate to high toxicity. However, these extracts have been tested only in terms of acute toxicity, thus chronic studies need to be performed in future. Although only a few studies on aquatic animals were mentioned, it can be said that the application of neem commercial pesticides should be handled with care and aqueous neem-based products should be encouraged.

Due to its pharmacological properties, the safety of *A. indica* extracts and/or isolated compounds should be evaluated, especially using mammalian models. Acute and subacute toxicity screenings of medicinal plants are a rapid way to assess its toxicological profile, providing an insight on safety/harmful aspects of compounds to evaluate. This paper reviewed a large number of studies about mammalian toxicity of neem. In terms of acute toxicity, the mode of administration had an important impact. Neem extracts/subproducts are nontoxic or less toxic when orally administered. Animals only revealed acute toxicity when treated by intramuscular injection or via the intraperitoneal route. In what concerns subacute and subchronic toxicity, neem extracts/subproducts’ toxicity is dose dependent. Higher concentrations were revealed to be more toxic regardless of the mode of administration. In terms of effects on reproduction and teratogenicity, some of the studies presented reported no effects, but most of them described changes in both the male and female reproductive systems.

Currently, little information is available on pharmacokinetic profiles (absorption, distribution, metabolism, and excretion (ADME)) of neem compounds. However, pharmacokinetic studies are important as a basis to determine toxicity and perform risk assessments, because they can provide data about concentrations in target organs and cells, uptake into blood, and distribution in the body. This represents an important future area of research, which should obtain ADME data available before toxicity tests. It is also important to keep in mind that, when performing in vivo tests with animals, the strategy should take into consideration the 3 R’s—refine, replace, and reduce: use as few animals as possible and refine the methods to minimize the suffering involved. Whenever possible, consider the replacement of in vivo studies with in vitro studies in cell cultures. In the particular case of *A. indica*, there are only a few studies evaluating its toxicity effects in vitro using normal cells. This represents a good opportunity for researchers, as further studies need to be conducted.

In conclusion, neem extracts present no or very low acute toxicity on mammalians. Subacute and subchronic toxicity on these animals can be eliminated if lower doses are used. Thus, due to these safety characteristics and their well-known pharmacological properties, neem tree and its compounds/extracts present a high commercial importance and can be considered serious candidates to new natural drugs therapies.

## Figures and Tables

**Table 1:** Toxicity of neem in different aquatic animals.

	Part of *A. indica*/Compound	Nature of the Extract	Test Animal	Dose	Duration	Effect Observed	References
Acute toxicity	Leaf	Aqueous	*Cirrhinus mrigala*	0.25, 0.50, 0.75, 1.0, 1.25, 1.50 ppm	24 h	LC_50_ = 1.035 ppm	[16]
	Root bark	Aqueous	*Artemia salina* L.	10, 100, and 1000 µg/mL	24 h	LC_50_ = 285.8 µg/mL	[17]
	Green callus (leaf)	Methanolic	*Danio rerio*	500, 1000, 2000, 3000, 4000, and 5000 µg/mL	5 d	LC_50_ = 4606 µg/mL (non-toxic)	[18]
	Azadirachtin	-	*Daphnia pulex*	0 to 0.5 µg/mL	48 h	LC_50_ = 0.328 µg/mL	[20]
			*Procambarus clarkii*	0.001, 0.01, 0.1, and 1 µg/mL	96 h	LC_50_ > 1 µg/mL	
	Azatin	-	*Daphnia pulex*	-	48 h	LC_50_ = 0.57 mg AZA/L (concentration based on azadirachtin equivalence)	[19]
	Neemix 4.5					LC_50_ = 0.68 mg AZA/L	
Acute toxicity	Neemix^TM^	-	*Daphnia pulex*	0 to 0.5 µg AZA/mL	48 h	LC_50_ = 0.07 µg AZA/mL	[20]
			*Culex quinquefasciatus*	0 to 1 µg AZA/mL	96 h	LC_50_ = 0.57 µg AZA/mL	
			*Callinectes sapidus*	0.25, 0.5, 1, 2 and 4 µg AZA/mL		LC_50_ = 1.15 µg AZA/mL	
			*Penaeus setiferus*	0.625, 1.25, 2.5, 5, and 10 µg AZA/mL		LC_50_ = 2.68 µg AZA/mL	
			*Palaemonetes pugio*			LC_50_ = 3.81 µg AZA/mL	
			*Procambarus clarkii*			LC_50_ = 6.60 µg AZA/mL	
	Bioneem^TM^	-	*Daphnia pulex*	0 to 0.5 µg AZA/mL	48 h	LC_50_ = 0.03 µg AZA/mL	
			*Culex quinquefasciatus*	0 to 1 µg AZA/mL	96 h	LC_50_ = 0.14 µg AZA/mL	
			*Palaemonetes pugio*	0.625, 1.25, 2.5, 5, and 10 µg AZA/mL		LC_50_ = 3.19 µg AZA/mL	
			*Procambarus clarkii*			LC_50_ = 4.71 µg AZA/mL	
			*Ceriodaphnia dubia*	0.01, 0.1, 1.0, and 10.0 mL/L	48 h	EC_50_ = 0.032 mL/L	[21]
Acute toxicity	Neem’s solution	-	*Daphnia magna*	-	48 h	LC_50_ = 17 µL/L	[22]
			*Cnesterodon decemmaculatus*		96 h	LC_50_ = 4.92 µL/L	
			*Limnoperna fortune* (larvae and adults)		72 h	LC_50_ = 8–249 µL/L	
	Bioneem oil	-	*Daphnia magna*	0.015, 0.031, 0.065, 0.125, 0.250, 0.50, 1.0, 2.0 mL/L	48 h	EC_50_ = 0.17 mL/L	[23]
			*Danio rerio*	0.16, 0.2, 0.32, 0.4, and 0.8 mL/L	96 h	LC_50_ = 0.22 mL/L	
Chronic toxicity			*Daphnia magna*	0.0106, 0.0212, 0.0425, 0.0850, 0.17 mL/L	21 d	Reduced number of neonates and inhibition of size	
	Bioneem^TM^	-	*Ceriodaphnia dubia*	0.001, 0.002, 0.004, 0.008, and 0.016 mL/L	7 d	Effects on mobility and reproduction	[21]
Genotoxicity	Azadiractin	-	*Oreochromis mossambicus*	0.0005% at 1 mL/100 g b.w.	96 h	Some somatic metaphase complements aberrations and proteotoxicity	[24]

**Table 2:** Acute toxicity of neem in different mammalians.

Part of *A. indica*	Nature of the Extract	Test Animal	Dose/Mode of Administration	Duration	LD_50_	Effect Observed	References
Leaves	Aqueous extract	Mice	200, 500, 1000, and 2500 mg/kg b.w./oral	1 d	>2500 mg/kg	No significant changes	[26]
		Mice	1250, 2500, and 5000 mg/kg b.w./oral	14 d	>5000 mg/kg	No significant changes	[27]
		Rats	0.05, 0.071, 0.084, 0.092, and 0.1 g/mL/intramuscular injection	2 d	6.2 mL/kg	Acute toxicity	[28]
		Rabbit	2.5 and 5 mg/kg b.w./oral	14 d	-	No significant changes	[38]
	Ethanolic extract	Mice	5 g/kg b.w./oral	7 d	>5 g/kg	No significant changes	[29]
		Mice	1000 mg/kg b.w./oral	1 d	>1000 mg/kg	No significant changes	[32]
		Rats	20, 200, or 2000 mg/kg b.w./oral	14 d	>2000 mg/kg	No significant changes	[30]
		Mice	50, 200, 400, 800, 1000, 1500, 2000, 3000, 4000, and 5000 mg/kg b.w./oral	1 d	>5000 mg/kg	No significant changes	[31]
		Rats	2000 and 5000 mg/kg b.w./oral	14 d	>5000 mg/kg	No significant changes	[33]
	Methanolic extract	Mice	10, 100, and 1000 mg/kg bw/intraperitoneal route	1 d	31.62 mg/kg	Highly toxic	[34]
Stem Bark	Methanolic extract	Mice	10, 100, and 1000 mg/kg b.w./intraperitoneal route	1 d	489.90 mg/kg	Moderately toxic	[34]
	Ethanolic extract	Rats	100, 200, 400, 800, 1600, 3200, and 6400 mg/kg b.w./intraperitoneal route	1 d	870 mg/kg	Acute toxicity	[35]
Seed	Aqueous extract	Rats	0.1, 0.15, 0.17, and 0.2 g/mL/intramuscular injection	2 d	9.4 mL/kg	Acute toxicity	[28]
Flowers	Methanolic extract	Rats	6, 9, and 12 g/kg b.w./oral	14 d	>12 g/kg	No significant changes	[36]
Neem oil	-	Mice	18.40, 23.00, 28.80, 36.00, and 45.00 g/kg b.w./oral	14 d	31.95 g/kg	No significant changes	[37]

**Table 3:** Subacute toxicity of neem in different mammalians.

Part of *A. indica*/Compound	Nature of the Extract	Test Animal	Dose/Mode of Administration	Duration	Effect Observed	References
Leaves	Aqueous extract	Rats	1000 mg/kg b.w./oral	28 d	No significant changes	[26]
	Aqueous extract (neem leaf preparation—NLP)	Mice	0.5, 1.0, or 2 units of NLP/injection	28 d	Death in higher concentration	[39]
	Neem leaf glycoprotein	Rats and Mice	25, 50, 100, and 200 μg/injection	28 d	No significant changes	[40]
	Hydroethanolic extracts	Rats	75, 150, and 300 mg/kg b.w./oral	28 d	No significant changes, except an increase aspartate amino transferase (ASAT) level	[33]
Stem Bark	Ethanolic extract	Rats	50, 100, 200, and 300 mg/kg b.w./oral	21 d	Alterations in biochemical parameters of toxicity	[41]
Nimbolide	-	Mice	1 mg/kg b.w./intraperitoneal route	21 d	No significant changes	[42]
Neem oil	-	Mice	177, 533, or 1600 mg/kg b.w./oral	28 d	No significant changes; higher dose group presented rough fur and loss of appetite in the last 2 weeks	[37]

**Table 4:** Subchronic toxicity of neem in different mammalians.

Part of *A. indica*/Compound	Nature of the Extract	Test Animal	Dose/Mode of Administration	Duration	Effect Observed	References
Vepacide (neem based-pesticide)	-	Rats	80, 160, and 320 mg/kg b.w./oral	45 d and 90 d	Alterations in lung, liver, and kidney tissues	[43]
Flowers	Methanolic extract	Rats	150, 750, and 1500 mg/kg b.w./oral	90 d	Slight toxicity	[36]
Neem oil	-	Mice	177, 533, and 1600 mg/kg b.w./oral	90 d	Lower doses had no damage on the serum biochemistry parameters and target organs were testicle, liver and kidneys	[44]

**Table 5:** Reproduction and teratogenicity effect of neem in different mammalians.

Part of *A. indica*/Compound	Nature of the Extract	Test Animal	Dose/Mode of Administration	Duration	Effect Observed	References
Leaves	Aqueous suspension	Rats	100 mg/day/oral	24 d	Ultrastructural changes in epithelial cells of cauda epididymis	[45]
	Aqueous suspension	Rats	100 mg/day/oral	48 d	Changes in Sertoli cells, followed by degeneration of germ cells	[46]
	Aqueous extract	Rats	125, 250, and 375 mg/kg b.w./oral	24 d	Alterations in biochemical parameters of testis and epididymis	[47]
	Aqueous extract	Rats	10 mg/g dry feed palate/oral	10 d	Oocytes apoptosis	[48]
	Ethanolic extract	Rats (pregnant)	65, 135, and 200 mg/kg b.w./oral	15 d	No significant changes	[49]
	Terpenoid extract	Rabbits (pregnant)	300 mg/kg b.w./oral	11 d	No significant changes	[50]
Stem Bark	Methanolic extract	Rats	150 mg/kg b.w./oral	45 d	Decrease in sperm count, viability and motility	[51]
Seed	Ethanolic extract	Rats	200 mg/oral	21 d	Alterations in the estrous cycle and partial block in ovulation	[52]
	Polar and non-polar fractions	Rats	3 and 6 mg/kg b.w./oral	18 d	Reduction of number of normal follicles	[53]
Flower	Ethanolic extract	Rats	1 g/kg or 10 mg/kg b.w./oral	21 d	Irregularity in estrous cycles, with extended diestrous stages	[54]
Wood ash	Aqueous extract	Mice	5, 50, and 100 mg/kg b.w./oral	35 d	Damage effects on sperms and testicular tissues	[55]
Azadirachtin	-	Rats	5, 25, and 50 mg/kg b.w./oral	21 d (in two generations)	No evidence of cumulative effects on postnatal development and reproductive performance over two generations	[56]
	-	Rats (pregnant)	1 mg/mL/oral	21 d	Defective intrauterine development and altered antioxidant/oxidative status during pregnancy	[57]
					No significant changes	[58]
Azadirachtin of neemix-4.5	-	Mice	0.9, 1.8, 3.0, 9.0, or 45.0 mg/kg b.w./subcutaneous injection	80 d or on days 7–12 of gestation	No significant changes	[59]
Azadirachtin-A	-	Rats	0.5, 1.0, and 1.5 mg/kg b.w./subcutaneous injection	24 d	Decrease in reproductive organs weight and increased abnormal sperms	[60]
Nimbolide	-	Rats	0.5, 1.0, and 1.5 mg/kg b.w./subcutaneous injection	24 d	Alterations in biochemical parameters of reproductive organs, sperm functional parameters, and fertility potency	[61]
Neem seed oil	-	Rats (pregnant)	1.2 mg/mL/oral	21 d	Defective intrauterine development and altered antioxidant/oxidative status during pregnancy	[57]
					Teratogenicity	[58]

## Data Availability

Data sharing not applicable.
